# Supplementary material for: WordCommentsAnalyzer: A windows software tool for qualitative research
Source: F1000Res. 2018 Sep 4;7:536. Originally published 2018 May 3. [Version 2] doi: 10.12688/f1000research.14819.2 (PMC6137411; doi:10.12688/f1000research.14819.2)
Supplement: Supplementary file 3 [file f1000research-7-17614-s0002.tgz › b2a79496-4ada-47c4-9682-a99ce867836d.zip!/Eight Iranian Health Journals/Salmand_Iranian Journal of Ageing _42.docx]

© Salmand_Iranian Journal of Ageing . This work is licensed under CC BY-NC 4.0 (https://creativecommons.org/licenses/by-nc/4.0/).

Volume & Issue: دوره 2 - شماره 4

Title: تأثیر یک دوره تمرینی ورزش در آب بر تعادل ایستا و پویای زنان سالمند

http://salmandj.uswr.ac.ir/article-1-52-fa.html

اهداف: ضعف تعادل از جمله عوامل خطرزای افتادن، آسیب دیدگی و یا حتی مرگ سالمندان به شمار می رود. این تحقیق با هدف ارزیابی تاثیر ورزش در آب بر تعادل ایستا و پویا زنان سالمند انجام شد. مواد و روش‌ها: 30 زن سالمند 55 تا 70 ساله، به‌طور داوطلبانه در دو گروه کنترل و تمرین در این تحقیق شرکت کردند. گروه تمرین در برنامه تمرینی ورزش در آب به مدت شش هفته، سه جلسه یک ساعته در هفته شرکت نمود. در هر دو گروه تعادل ایستا و پویا قبل و بعد از دوره درنظر گرفته شده، اندازه گیری شد. شاخص‌های نوسانات قامتی شامل میانگین تغییرات مرکز فشار (COP) و سرعت تغییرات مرکز فشار (COP VEL) در جهت جانبی داخلی (ML) و قدامی خلفی (AP)، در حالت ایستاده روی یک پا، به عنوان شاخص تعادل ایستا و تست‌های پیش‌روی رو به جلو (FRT)، تست پیش‌روی جانبی در سمت راست (FRRT) و تست پیش‌روی جانبی در سمت چپ (FRLT) به عنوان شاخص تعادل پویا در نظر گرفته شد. از تست تی همبسته جهت مقایسه تغییرات درون گروهی، و از تست تی مستقل جهت مقایسه شاخص‌های تعادل بین دو گروه پس از شش هفته در سطح معنی‌داری 0/05 استفاده شد. یافته‌ها: نتایج نشان داد که یک دوره تمرینی، باعث بهبود در نتایج تست‌های پیش‌روی روبه جلو، و پیش‌روی در سمت چپ گردید. ضمن اینکه نوسانات قامتی شامل جابه‌جایی های مرکز فشار و سرعت تغییرات مرکز فشار تنها در جهت جانبی داخلی در گروه تمرین پس از شش هفته ورزش در آب بهبود یافت، اما تغییرات معنی‌داری در میانگین و سرعت تغییرات مرکز فشار در جهت قدامی خلفی مشاهده نشد. همچنین اختلاف معناداری در میانگین و سرعت تغییرات مرکز فشار و تست پیش‌روی عملکردی روبه جلو و سمت چپ بین دو گروه بعد از تمرین مشاهده گردید (P≤0/05). نتیجه‌گیری: با توجه به یافته های تحقیق چنین می توان گفت اعمال برنامه تمرین در آب برای سالمندان (در محیط نامتعادل و بی ثبات) سیستم‌های فیزیولوژیک درگیر در تعادل را به چالش می‌کشد که نتیجه آن بهبود در وضعیت تعادل ایستا به ویژه در جهت جانبی داخلی و پویا با افزایش فاصله دستیابی روبه جلو و سمت چپ، و در نتیجه، کاهش خطر افتادن است.

Title: تبیین دیدگاه سالمندان پیرامون پدیده احساس تنهایی: یک پژوهش کیفی پدیدارشناسی

http://salmandj.uswr.ac.ir/article-1-53-fa.html

اهداف: هدف این مطالعه تبیین دیدگاه سالمندان نسبت به مفهوم پدیده احساس تنهایی بود. مواد و روش‌ها: این مطالعه به روش تحقیق کیفی با رویکرد پدیدارشناسی تاویلی انجام شد. به این منظور مصاحبه های بدون ساختار با 13 سالمند 68 تا 87 ساله ساکن در شهر تهران صورت گرفت که در طی آن سالمندان تجربیات خود را در خصوص این پدیده بیان نمودند. داده های به دست آمده از این مصاحبه ها با استفاده از روش پدیدارشناسی ون مانن مورد تجزیه و تحلیل قرار گرفت. یافته‌ها: 3 تم از یافته‌ها استخراج شد که می توانند دیدگاه سالمندان از پدیده احساس تنهایی را به تصویر کشند. این تم ها عبارتند از: حس درد و رنج، فقدان و محرومیت، و راه های جبران. نتیجه‌گیری: یافته‌های این مطالعه توانست تصویری روشن از دیدگاه سالمندانی که همگی به نوعی پدیده احساس تنهایی را تجربه نموده بودند ارایه دهد. تجربیات سالمندان از این پدیده نشان داد که آن ها احساس تنهایی را یک حس دردناک و رنج آور می دانند که در نتیجه فقدان های موجود در زندگی بروز می نماید و آنان در ضمن ایجاد ارتباط با خدا با یادآوری خاطرات دوران های مختلف زندگی و ایجاد ارتباطات جدید در جهت رویارویی با این پدیده تلاش می کنند. این یافته‌ها مفاهیم مهمی را در عرصه ارایه مراقبت به سالمندان آشکار ساخته و می تواند توانایی ارایه دهندگان مراقبت های بهداشتی- درمانی و توانبخشی را برای بررسی، پیشگیری و مداخلات مراقبتی در خصوص این پدیده افزایش دهد.

Title: الگوی مصرف دارو در افراد 55 سال به بالای ساکن شهر تهران

http://salmandj.uswr.ac.ir/article-1-55-fa.html

اهداف: یکی از بخش‌های جمعیتی در حال رشد جامعه ما جمعیت افراد سالمند است. سالمندان نسبت به سایر گروه‌های سنی بزرگترین مصرف‌کنندگان دارو هستند. بیماری‌های مزمن و مصرف داروهای متعدد به همراه تغییرات فیزیولوژیکی افزایش سن، باعث آسیب‌پذیری سالمندان نسبت به آثار سوء‌ دارودرمانی می شوند. هدف این مطالعه تعیین الگوی مصرف دارو و عوامل موثر بر آن در افراد 55 سال به بالای ساکن تهران است. مواد و روش‌ها: در این مطالعه توصیفی- تحلیلی که اطلاعات به‌صورت مقطعی جمع‌آوری شد 400 نفر نمونه با روش دو مرحله‌ای و تصادفی از طریق مصاحبه و تکمیل پرسش‌نامه در درب منازل بررسی شدند. برای آنالیز داده‌ها از آمار توصیفی و استنباطی (آزمون‌های Chi-square و Fisher) در محیط SPSS استفاده گردید. یافته‌ها: میانگین مصرف روزانه دارو 1/9±3/4 قلم در روز بود. 40درصد نمونه ها بیش از سه قلم دارو در روز مصرف و 35درصد برای یک بیماری خاص به پزشکان متعدد مراجعه و 19درصد دچار عارضه جانبی شده‌اند. آسپیرین، آتنولول و پروپرانولل پرمصرف‌ترین داروها بودند. الگوی مصرف داروهای پرمصرف به‌جز آسپیرین در دو جنس، گروه‌های مختلف سنی، تحصیلی و مال تفاوت معنی‌داری نداشت. پرمصرف‌ترین دسته داروهای اصلی داروهای قلبی عروقی (35درصد)، سیستم اعصاب مرکزی (25درصد)، و هورمون‌ها (9درصد) و پزشکان اصلی‌ترین منبع کسب اطلاعات مربوط به داروها بودند. نتیجه‌گیری: ارائه برنامه‌های آموزشی برای پزشکان و داروسازان و خانواده‌ها برای اصلاح فرهنگ تجویز و مصرف دارو ضروری است.

Title: جداسازی باکتری‌های فستی‌دیوس (Fastidious) در نمونه‌های ادراری زنان سالمند

http://salmandj.uswr.ac.ir/article-1-56-fa.html

اهداف: هدف تحقیق حاضر این بوده است تا با استفاده از روش جداسازی باکتری‌های Fastidious در نمونه‌های ادراری زنان سالمند که با روش معمول نتایج کشت آن‌ها منفی است ولی بیماران دارای علایم عفونت هستند در تشخیص و درمان آن‌ها موثر واقع گردد. مواد و روش‌ها: این تحقیق در بیمارستان امام خمینی انجام یافته و مسئولیت جداسازی و تشخیص باکتری‌های فستی دیوس از 1075 نمونه ادراری زنان سالمند بالاتر از 60 سال را بر عهده داشته است که با تغییر در محیط‌های کشت و شرایط انکوبه‌کردن و میزان گازها توانسته است باکتری‌های فستی‌دیوس را جداسازی نماید. همچنین از تعداد لکوسیت در نمونه ابتدایی برای تعیین وجود چرک در ادرار استفاده گردیده ضمناً مطالعه پرونده‌های پزشکی به‌منظور تعیین نشانه‌ها و مصرف آنتی‌بیوتیک انجام و در پایان کلیه نتایج با یکدیگر تطبیق و با گروه شاهد مورد ارزیابی قرار می‌گرفتند. یافته‌ها: پس از کشت هوازی و شبانه 1075 نمونه ادراری، 383 مورد مثبت، 99 مورد دارای شمارش پایین، 159 مورد به صورت مخلوط و 434 مورد منفی بود. از 434 مورد منفی در روش مذکور، با استفاده از تکنیک جداسازی باکتری‌های فستی دیوس 147 مورد دارای باکتری‌های فستی دیوس در این تحقیق بود که بیش از 50درصد آنان با نشانه های عفونت همراه بود که اختلاف معنی‌داری را نشان می‌دهد. نتیجه‌گیری: برای جداسازی و تشخیص عوامل عفونت ادراری زنان سالمند بیمار که کشت هوازی و شبانه آن‌ها منفی است به‌خصوص اگر همراه با حضور نشانه‌ها باشد کشت نمونه آن‌ها در محیط‌های اضافی و انتخابی همراه با تغییر در شرایط انکوباسیون برای جداسازی باکتری‌ها الزامی است.

Title: میزان رضایتمندی از سمعک در زندگی روزمره سالمندان کم‌شنوا

http://salmandj.uswr.ac.ir/article-1-57-fa.html

اهداف: هدف از مطالعه حاضر، بررسی میزان رضایتمندی از سمعک و عوامل موثر بر آن در زندگی روزمره سالمندان کم‌شنوا بود. مواد و روش‌ها: در مطالعه مقطعی حاضر، 32 سالمند استفاده کننده از سمعک با کم‌شنوایی حسی عصبی متوسط تا شدید (میانگین سن 9/8±63/5 سال) مراجعه‌کننده به کلینیک شنوایی بخش خصوصی (کلینیک شنوایی‌شناسی آلتون شنوا، مرکز توانبخشی شنوایی نیوشا، کلینیک شنوایی شناسی گلریز) جهت پاسخ‌دهی به نسخه فارسی پرسش‌نامه "سنجش رضایتمندی از سمعک در زندگی روزمره" انتخاب شدند. این پرسش‌نامه، میزان رضایتمندی را از طریق یک امتیاز کلی و چهار زیر آزمون به شکل کمّی نشان می دهد. بر اساس فرم تاریخچه‌گیری، وضعیت اقتصادی- اجتماعی افراد در حد متوسط قرار داشت. یافته‌ها: متوسط کل امتیاز در نسخه فارسی پرسش‌نامه حاضر، 0/57±4/24 (حداقل 2/24 و حداکثر 5/12) بود که میزان رضایت در حد متوسط را نشان می‌دهد. متوسط امتیاز در زیرآزمون «تأثیرات مثبت» 0/83±4/33 (رضایت متوسط تا نسبتاً زیاد) زیرآزمون «خدمات و هزینه‌ها» 0/84±3/60 (رضایت نسبتاً پایین)، زیرآزمون «ویژگی‌های منفی» 0/94±4/05 (رضایت متوسط)، و در زیرآزمون «خودپنداری» 1/25±5/00 (رضایت نسبتاً زیاد) بود. بین سطح رضایتمندی با میزان استفاده از سمعک، ساعات استفاده از سمعک و سطح تحصیلات ارتباط معناداری مشاهده شد (P<0/05). اما بین دو جنس در سطح رضایتمندی، تفاوت معناداری وجود نداشت (P<0/0566). نتیجه‌گیری: نتایج رضایت سالمندان استفاده‌کننده از سمعک، نسبت به سایر مطالعات قبلی در این زمینه، سطح پایین‌تری را نشان می‌دهد. با بررسی عوامل مرتبط با رضایتمندی از سمعک، به نظر می‌رسد عواملی همچون استفاده از فناوری روز، ارزیابی نیازهای ارتباطی و شنوایی (پیش از تجویز سمعک و پس از آن) و مشاوره دقیق درمورد توانایی‌ها و محدودیت‌های این پروتز در کنار بهبود ارائه خدمات عمومی بتواند در افزایش رضایت استفاده‌کنندگان از سمعک، تاثیر داشته باشد.

Title: تأثیر برنامه ورزشی بر کیفیت زندگی سالمندان شهرستان شهرکرد

http://salmandj.uswr.ac.ir/article-1-58-fa.html

اهداف: سرعت رشد جمعیت سالمندان در کشورهای در حال توسعه بسیار زیاد است، بعلاوه سطح کیفیت زندگی در سنین سالمندی به دلایل متعددی، به‌خصوص، به‌علت کمبود تحرک فیزیکی و فعالیت ورزشی کاهش می‌یابد. این مطالعه با هدف بررسی میزان تاثیر برنامه ورزشی بر کیفیت زندگی سالمندان شهرستان شهرکرد انجام گرفت. مواد و روش‌ها: در این مطالعه نیمه تجربی که در شهرستان شهرکرد در سال 1386 انجام گرفت، 60 نفر سالمند با میانگین سنی 6/96±68/63 در گروه آزمون و 10/65±68/03 در گروه شاهد در این مطالعه شرکت داشتند. این افراد دارای توانایی انجام فعالیت ورزشی و انجام کارهای معمول و روزانه بدون وابستگی به دیگران و فاقد بیماری‌های سیستمیک و محدودکننده، بودند. و به‌صورت نمونه‌گیری دردسترس انتخاب و به‌طور تصادفی به دو گروه آزمون و شاهد تقسیم شدند، در گروه آزمون برنامه ورزشی به مدت 60 جلسه اجرا گردید. سطح کیفیت زندگی هر دو گروه، قبل از مداخله و در پایان مداخله به‌وسیله پرسش‌نامه لیپارد ارزیابی گردید. جهت تجزیه و تحلیل آماری داده ها از آزمون‌های تی و آزمونهای کای دو و من ویتنی استفاده گردید. یافته‌ها: با به‌کارگیری برنامه ورزشی پیش‌رونده عضلانی سطح کیفیت زندگی افراد به طور معنی‌داری نسبت به قبل از اجرای برنامه افزایش یافت (P<0/05) و این در حالی است که در مدت زمان مشابه، در گروهی که برنامه ورزشی را تمرین نمی‌کردند تغییر معنی‌داری در ابعاد کیفیت زندگی آنان ایجاد نشده است. نتیجه‌گیری: با توجه به یافته‌های حاصل از این پژوهش می‌توان نتیجه‌گیری نمود که با به‌کارگیری برنامه ورزشی منظم، مداوم و دارای پذیرش خوب از سوی این گروه سنی، می توان سطح کیفیت زندگی را در ابعاد مختلف در این افراد افزایش داد و راه را برای سالم پیرشدن و برخوردار بودن از سلامتی در این دوره هموار نمود.

Title: عوامل تسهیل‌گر و بازدارنده مراقبت خانوادگی از سالمندان آسیب‌پذیر در منزل: تجربه زنان مراقب

http://salmandj.uswr.ac.ir/article-1-59-fa.html

اهداف: با افزایش جهانی جمعیت سالمندان، مراقبت خانوادگی از اجزای مهم مراقبت طولانی مدت به حساب آمده و زنان به عنوان اصلی‌ترین مراقبان خانوادگی، عهده‌دار این امر شده‌اند. مطالعه کیفی عوامل تسهیل‌گر و بازدارنده‌ای که در فرایند مراقبت خانوادگی موثر هستند، می‌تواند تصویر روشن‌تری از نیاز و تقاضای مراقبین فراهم کرده و بدین ترتیب منجر به تدبیر موثرتر و سیاست‌گذاری کیفی‌تر برای ارتقای سلامت زنان مراقبت دهنده و سالمندان گردد. مواد و روش‌ها: بر اساس مطالعه کیفی گراندد تئوری، تجربیات زیسته 12 مراقب زن از طریق مصاحبه عمیق نیمه‌ساختاری و مشاهده در عرصه جمع‌آوری و بازنویسی گردید و بر اساس رویکرد استراوس و کوربین 1998 تحت کدگذاری باز، محوری و انتخابی قرار گرفت. جهت حصول دقت در مطالعه از روش‌های مرور و تایید مطالب توسط شرکت‌کنندگان، اعتبار پژوهشگر، وابستگی یافته‌ها، بررسی طولانی و مشاهده مداوم، تلفیق در روش‌های جمع‌آوری داده‌ها و نظرات تکمیلی همکاران استفاده شد. یافته‌ها: مراقبین در طول تجربه فرایند مراقبت از سالمند عضو خانواده خود، با دو دسته عوامل تسهیل‌گر و بازدارنده روبه‌رو بودند. کدهای به‌دست‌آمده در 9 زیر طبقه شامل آگاهی از نحوه مراقبت، ویژگی‌های فردی مراقب و سالمند، تجربه بار مراقبتی، سابقه ارتباطی موثر بین مراقبت و سالمند، شبکه حمایتی خانوادگی، مشارکت خانوادگی، سیستم حمایتی رسمی، زمینه مراقبت در منزل و سیستم‌های بهداشتی و مراقبتی رسمی ویژه سالمندان طبقه‌بندی شدند که هر یک حسب ماهیت سبب تسهیل و یا ممانعت در فرایند مراقبت خانوادگی می‌شدند. بر اساس ویژگی، زیر طبقات در 3 طبقه اصلی عوامل فردی، خانوادگی و خارجی دسته بندی شدند. نتیجه‌گیری: عوامل تسهیل‌گر و بازدارنده بنا بر ماهیت خود با ایجاد سهولت و یا صعوبت در ارائه مراقبت به سالمند توسط زنان مراقب، می‌توانند بر چگونگی تجربه آنان موثر باشند. بر اساس یافته‌های حاصل از تجارب شرکت‌کنندگان تقویت عوامل تسهیل‌گر از طریق جلب مشارکت سایر اعضای خانواده، حمایت روانی و اجتماعی مراقبان، ارتقای دانش مراقبین از طریق آموزش نحوه مراقبت، تامین امکانات و تسهیلات، تمهید بستر مناسب مراقبت و طراحی سیستم‌های مراقبتی کارآمد دوستدار سالمند و خانواده سبب توفیق بیشتر مراقبین در ارائه مراقبت به سالمند عضو خانواده می‌گردد.

Title: امنیت اجتماعی و آسیب‌شناسی سالمندان در شهر تهران: پژوهشی از دیدگاه جامعه‌شناسی

http://salmandj.uswr.ac.ir/article-1-60-fa.html

اهداف: پژوهش حاضر در تلاش است تا وضعیت زندگی سالمندان را در شهر تهران با تاکید بر امنیت اجتماعی و آسیب‌شناسی آن‌ها، مورد مطالعه قرار دهد. در این تحقیق، ابعاد مختلف زندگی مادی، اجتماعی، سلامت و وضعیت تغذیه سالمندان مورد مطالعه قرار می‌گیرد. درحالی‌که جامعه آماری این پژوهش سالمندان شهر تهران است. در آن حقوق انسانی، شأن سالمندان، و افت نسبی امنیت اجتماعی این جمعیت مورد مطالعه و ارزیابی قرار می‌گیرد. از جمله اهداف عمده این پژوهش پی‌بردن به ابعاد مختلف زندگی سالمندان و گروه‌های سنی است، که به‌تدریج توان و خود اتکایی جسمی و عقلی خود را از دست می دهند، و در نتیجه میزان وابستگی آن‌ها به سایرین و خدمات گوناگون افزایش می‌یابد. مواد و روش‌ها: جامعه آماری این پژوهش شهر تهران در نظر گرفته شده است. در آن، با تاکید بر عملیات میدانی و عملی، ضمن مراجعه به 500 نفر سالمند به طور تصادفی، اطلاعات مورد نظر از طریق پرسش‌نامه اخذ گردیده است. یافته‌ها: اطلاعات حاصله از تحقیق حاضر بیانگر این واقعیت است که هرم سنی سالمندان در بین جمعیت نمونه از سنین شصت سالگی به بعد سریعا در حال باریک‌شدن است؛ بنحوی که از این مرز سنی به بعد، فقط 60 درصد سالمندان دارای همسر بوده‌اند. یافته‌های تحقیق خود بیانگر انعکاسی روشن از موقعیت اقتصادی، اجتماعی، درمانی، حمایت‌های عاطفی، ارتباط فرزندان با آن‌ها و... است. همین‌طور، در این تحقیق انواع سرگرمی‌ها و وضعیت مسکن سالمندان نیز مورد جست‌وجو قرار گرفته است. نتیجه‌گیری: بین سایر نتایج، تحقیق حاضر به این نتیجه دست یافت که سالمندان جدید یا متولدین سالهای 1320 به بعد، در شرایط اجتماعی، اقتصادی و فرهنگی عصر حاضر، در مقایسه با سالمندان نسل‌های پیش از آن، به‌ویژه در شهر تهران دارای نیازها و انتظارات جدیدی هستند، که باید توسط موسسات ذیربط پاسخ داده شوند.
